# Upadacitinib in Biologic-Experienced Inflammatory Bowel Disease: Real-World Efficacy, Safety, and Laboratory Outcomes

**DOI:** 10.3390/medicina61091692

**Published:** 2025-09-18

**Authors:** Osman Özdoğan, Serkan Yaraş

**Affiliations:** Department of Internal Medicine (Gastroenterology), Faculty of Medicine, Mersin University, 33343 Mersin, Turkey

**Keywords:** Upadacitinib, ulcerative colitis, Crohn’s disease, real-world evidence, safety, laboratory parameters

## Abstract

*Background and Objectives*: Upadacitinib is a recently approved Janus kinase (JAK) inhibitor for the treatment of inflammatory bowel disease (IBD). Although it has been adopted for clinical use, there are limited real-world data regarding its efficacy and safety, as well as its effects on laboratory parameters. In our study, we aimed to evaluate these outcomes in patients with moderate-to-severe Crohn’s disease (CD) and ulcerative colitis (UC) who had previously failed biologic agents. *Materials and Methods*: This retrospective cohort study included 22 UC and 19 CD patients who received Upadacitinib for at least six months, where efficacy was assessed at pretreatment and at 3 and 6 months. We used the Harvey–Bradshaw Index (HBI) for CD and the partial Mayo score (PMS) for UC to define clinical response and remission. *Results*: All patients were biologic-experienced, with a substantial portion (47%) having previously failed at least two different agents. At six months, the persistence rates were 78.3% for CD and 88% for UC. The clinical response rates were 72.7% for CD and 83.3% for UC, while the clinical remission rates were 50% and 58.3%, respectively. Fecal calprotectin and C-reactive protein levels significantly improved (*p* < 0.001). Total, LDL, and HDL cholesterol levels increased, but triglyceride levels and the LDL/HDL ratio remained unchanged. Biochemical parameters, including glucose, HbA1c, and thyroid, kidney, and liver function tests, remained within normal limits. No clinically significant changes were observed in hemogram parameters, and no serious adverse events, embolisms, major cardiovascular events (MACEs), or deaths occurred. *Conclusions*: Upadacitinib is effective in biologic-unresponsive IBD patients. While it increases cholesterol levels, it does not alter the LDL/HDL ratio and does not demonstrate a negative effect on glucose metabolism. Further multicenter, longer-term studies are needed on this topic.

## 1. Introduction

Upadacitinib (UPA) is an oral Janus kinase (JAK) inhibitor that modulates the JAK–STAT signaling pathway, which plays a central role in the pathogenesis of several inflammatory diseases, including rheumatoid arthritis (RA), ankylosing spondylitis (AS), and atopic dermatitis (AD) [1]. In recent years, Upadacitinib has also received indications for ulcerative colitis (UC) and Crohn’s disease (CD) and is now widely used worldwide [2,3]. Data from phase studies—including U-ACHIEVE and U-ACCOMPLISH for UC and U-EXCEED and U-ENDURE for CD—have demonstrated its efficacy [3,4,5].

Upadacitinib is a drug in the JAK inhibitor group. While JAK inhibitors are effective in treating various inflammatory diseases, they also have certain side effects. Tofacitinib, another JAK inhibitor, was first approved for the treatment of RA in 2012 [6]. Numerous studies have been conducted on JAK inhibitors, which have been used for many years in autoimmune diseases other than inflammatory bowel disease (IBD), and their side effects are well documented [7,8,9]. These studies have primarily evaluated JAK inhibitors other than Upadacitinib and have focused on serious adverse effects such as venous thromboembolism (VTE) and major adverse cardiac events (MACEs), as well as the development of opportunistic infections, lymphoma, and skin cancer.

Unlike other JAK inhibitors, Upadacitinib has greater selectivity for JAK1 than for JAK2, JAK3, and tyrosine kinase 2 (TYK2) [10]. In phase studies of Upadacitinib in patients with IBD, the rates of serious adverse events (AEs), such as MACEs or venous thromboembolism, were similar to those in the placebo group. However, significant increases were observed in liver function test values, cholesterol levels, and enzymes such as creatine phosphokinase compared to the placebo group [3,4,5].

Real-world data are almost nonexistent, apart from phase studies examining changes in efficacy, side effects, and laboratory parameters in IBD patients receiving Upadacitinib [11,12]. The present study evaluated the clinical outcomes and side effects of Upadacitinib in IBD patients, with a particular focus on changes in lipid profile, glucose levels, kidney and liver function tests, vitamin levels (including ferritin, vitamin B12, and folic acid), thyroid function tests (TSH and FT4), and hematologic parameters such as hemoglobin, neutrophil and lymphocyte counts, and platelet counts.

## 2. Materials and Methods

### 2.1. Data Source and Study Population

This retrospective cohort study evaluated the efficacy, side effects, and, specifically, the effects of Upadacitinib on laboratory parameters in patients with moderate-to-severe CD or UC. This study was approved by the Institutional Ethics Committee (protocol no. 2025/750), and all participants provided written informed consent.

This study included patients aged 18 years and older who had previously used biologics but lost response, had recently started Upadacitinib, or were undergoing regular follow-up. Patients were excluded if they had a history of cardiovascular disease, stroke, pulmonary embolism, deep vein thrombosis, liver cirrhosis, or renal failure (GFR < 30 mL/min/1.73 m^2^). Exclusion criteria also included patients who initiated new medications or underwent significant treatment changes during the study period (e.g., introduction of a new statin or diabetes medication). Other exclusions were pregnant or breastfeeding women, patients with chronic infections such as hepatitis B or HIV, and those with active infections like pneumonia or abscesses. Patients taking vitamin supplements such as iron, vitamin B12, or folic acid were also excluded from this study. Additionally, patients receiving immunomodulatory therapy (e.g., azathioprine) were required to discontinue it before starting Upadacitinib.

A total of 53 patients who presented to our clinic between 25 June 2024 and 31 December 2024 were enrolled in this study. Upadacitinib was administered at an induction dose of 45 mg daily for the first 3 and 2 months for CD and UC, respectively. All UC patients completed the standard 2-month induction period, and none required an extended induction with Upadacitinib. Maintenance therapy was continued at 15 mg daily for four patients aged ≥60 years and at 30 mg daily for the remaining patients.

Five patients were excluded from this study due to an inability to attend regular follow-up visits, and two patients discontinued treatment during the induction phase because of adverse effects such as nausea and vomiting. For the evaluation of Upadacitinib persistence, clinical remission, and clinical response, data from an additional five patients were included: two who did not respond to induction therapy (one with UC and one with CD) and three who lost response during maintenance therapy (one with UC and two with CD). These five patients were excluded from analyses of other outcomes.

The final study cohort included 22 patients with UC and 19 with CD. For the patients with Crohn’s disease, location and behavior were classified according to the Montreal classification [13]. Ulcerative colitis was categorized into three groups according to disease extent: left-sided colitis, extensive colitis, and pancolitis.

### 2.2. Data Collection

Patients were assessed at three time points: baseline (before starting Upadacitinib), and at 3 and 6 months of treatment. All laboratory assessments were performed after at least 8 h of fasting and included measurements of glucose, glycated hemoglobin (HbA1c), urea, creatinine, aspartate aminotransferase (AST), alanine aminotransferase (ALT), gamma-glutamyl transferase (GGT), alkaline phosphatase (ALP), total bilirubin (TB), albumin, thyroid-stimulating hormone (TSH), free thyroxine (FT4), calcium, total cholesterol (TC), high-density lipoprotein cholesterol (HDL-C), low-density lipoprotein cholesterol (LDL-C), triglycerides, ferritin, vitamin B12, folic acid, C-reactive protein (CRP), and complete blood counts. All tests were performed in the hospital laboratory using Roche Cobas C501 and Beckman Coulter LH 780 analyzers.

Fecal calprotectin (FCAL) was measured by enzyme immunoassay (EIA) (Calprotectin [MRP 8/14] [Stool] ELISA, Immundiagnostik AG, Stubenwald-Allee 8a, 64625 Bensheim, Germany) with a linear measurement range of 13–840 μg/g.

Adverse events and clinical status were assessed at each visit, and abdominal ultrasonography was performed for all patients to evaluate intestinal activity. Disease severity was determined using the Harvey–Bradshaw Index (HBI) in CD patients and the partial Mayo score (PMS) in UC patients. CD: Clinical response constituted an HBI improvement of ≥3 points, and clinical remission correlated with an HBI < 5. UC: Clinical response was indicated by a PMS improvement of ≥2 points, and clinical remission a PMS < 2. Active disease was defined by CRP > 5 mg/L and/or clinical, endoscopic, or radiographic evidence of activity, and/or FCAL ≥ 250 μg/g.

### 2.3. Statistical Analysis

Descriptive statistics for demographic and laboratory parameters are expressed as the mean ± standard deviation. The Shapiro–Wilk test was applied to assess the normality of distributions. For comparisons of laboratory parameters at baseline, 3 months, and 6 months, paired *t*-tests and repeated-measures ANOVA were used for normally distributed data, while the Wilcoxon signed-rank test and Friedman test were applied to non-normally distributed data. The effect size of the treatment was assessed by calculating change ratios (percentage change). A *p*-value of <0.05 was considered statistically significant. Statistical analyses were performed using SPSS (Statistical Package for the Social Sciences) version 25.0 (IBM Corp., Armonk, NY, USA).

## 3. Results

### 3.1. Baseline Characteristics

The study cohort comprised 41 patients, of whom 15 (36.6%) were female. The mean age of our patients, which ranged from 21 to 69 years, was 43.6 ± 14.3 years, and the mean disease duration was 8.1 ± 5.1 years (ranging from 2 to 24 years). UC patients were older than CD patients (UC: 48 ± 15.4 years vs. CD: 38.1 ± 10.7 years), but the mean disease duration was similar (UC: 7.8 ± 4.4 years vs. CD: 8.5 ± 5.9 years). Seventeen (41.5%) patients were current smokers. Before starting Upadacitinib, 46.3% of our patients (*n* = 19) had previously used more than one biologic. The most common site of involvement in CD was the ileum (L1), while extensive colitis was the most common type in UC patients. Seven (36.9%) of the CD patients had ankylosing spondylitis (AS), while only one patient in the UC group had AS. Only one patient had diabetes. One patient had Bartter syndrome, which, aside from hypokalemia, did not affect kidney function. Compared to CD patients, pretreatment fecal calprotectin and CRP levels were higher in UC patients, while albumin levels were slightly lower. Patient demographics are summarized in Table 1.

Upadacitinib has been in use in Turkey for over a year. Before its introduction, only anti-TNF (Infliximab, Adalimumab), anti-integrin (Vedolizumab), and anti-interleukin (IL)-12/23 (Ustekinumab) molecules were used as biological agents in IBD patients. Except for one UC patient, all 40 patients (97.6%) were anti-TNF-experienced. The most commonly used biologic was infliximab, which was used by 26 patients (63.4%). Prior use of azathioprine was noted in 36 patients (87.8%), representing a large majority of the study cohort. Methotrexate is not the most preferred drug for IBD treatment in our country and at our clinic. A total of 11 patients (26.8%; 6 with CD, 5 with UC) who began Upadacitinib induction therapy were also started on concomitant corticosteroids (Table 2).

### 3.2. Upadacitinib and Efficacy

As previously mentioned, two patients (one with UC, one with CD) were excluded from this study due to a lack of response to induction therapy, and three patients (one with UC, two with CD) were excluded due to a loss of response to maintenance therapy. Two additional patients (one with UC, one with CD) were also excluded because they were unable to use the medication due to side effects such as nausea and vomiting. At the end of the sixth month, one patient with CD developed a perianal abscess, leading to a loss of efficacy. Based on these data, the persistence rates of Upadacitinib at the end of the sixth month were 78.3% for CD and 88% for UC.

**Figure 1:**
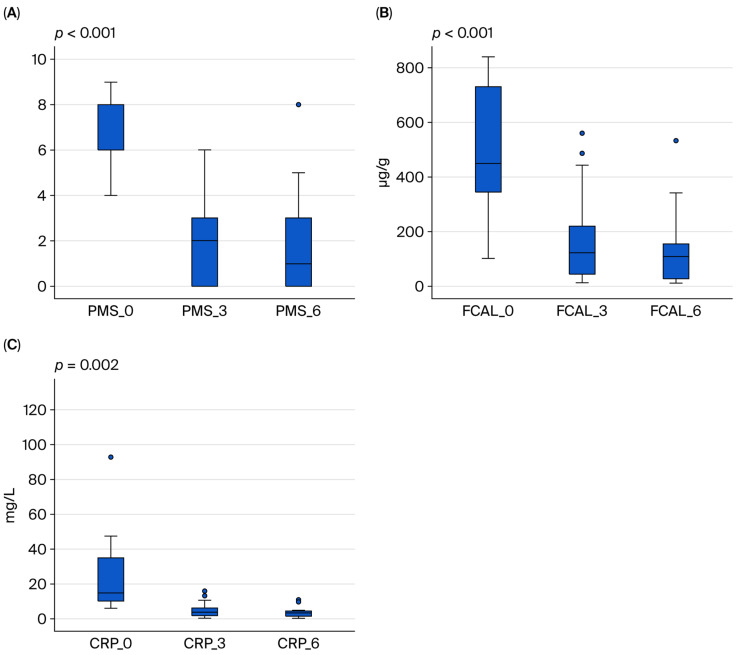
Changes in (**A**) partial Mayo score (PMS), (**B**) fecal calprotectin (FCAL), and (**C**) CRP levels in patients with ulcerative colitis before Upadacitinib treatment and at 3 and 6 months.

According to the partial Mayo score (PMS) calculated before Upadacitinib treatment, 20 (90.9%) of 22 UC patients were in the moderate-to-severe group. After 6 months of treatment, this number decreased to two (9.1%) (Table 3). Including the one patient who did not respond to induction therapy and the one who lost response to maintenance therapy, the clinical response rate in UC patients receiving Upadacitinib was calculated as 83.3%, and the clinical remission rate was 58.3% at the end of the sixth month. The clinical response rate in maintenance therapy without steroids was 72.8%. Furthermore, the proportion of UC patients with FCAL levels greater than 250 µg/g was 81.8% before Upadacitinib, which decreased to 18.2% by the sixth month of treatment. While 100% of UC patients had CRP values greater than 5 mg/L before Upadacitinib treatment, this rate decreased to 18.2% at the end of the sixth month of treatment with Upadacitinib (*p* < 0.05) (Table 3, Figure 1).

**Figure 2:**
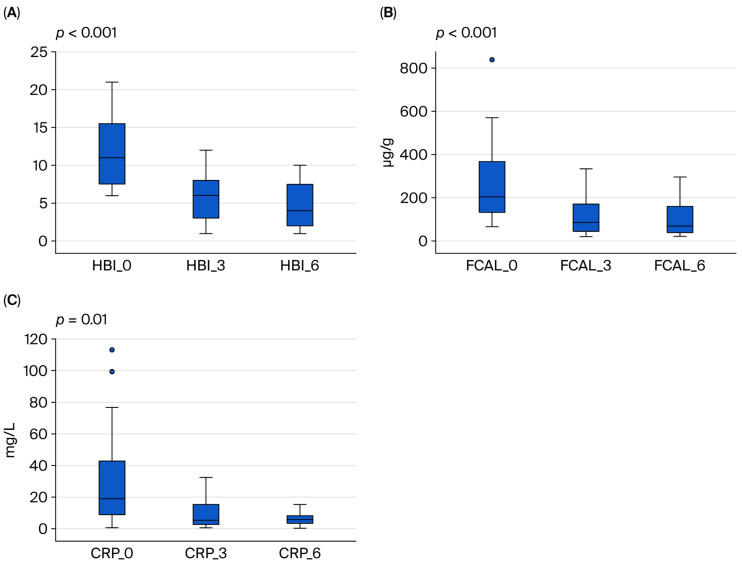
Changes in (**A**) Harvey–Bradshaw Index (HBI), (**B**) fecal calprotectin (FCAL), and (**C**) CRP levels in patients with Crohn’s disease before and at 3 and 6 months of Upadacitinib treatment.

According to the Harvey–Bradshaw Index (HBI) calculated before Upadacitinib treatment, 14 (73.7%) of the CD patients were in the moderate-to-severe group. At 6 months of Upadacitinib treatment, the rate of patients with an HBI < 5 was found to be 57.9% (Table 4). When we included a total of three patients who did not respond to induction therapy and lost response during maintenance therapy, the clinical response rate at the end of the sixth month in CD patients receiving Upadacitinib was 72.7%. In contrast, the clinical remission rate was calculated as 50%. The clinical response rate in maintenance therapy without steroids was 63.2%. The rate of patients with FCAL levels exceeding 250 µg/g was 42.1% at baseline, and this rate significantly decreased to 10.2% at 6 months of treatment. While 94.7% of CD patients had CRP values greater than 5 mg/L before Upadacitinib treatment, this rate decreased to 47.4% at the end of the sixth month of treatment with Upadacitinib (*p* < 0.05) (Table 4, Figure 2).

### 3.3. Upadacitinib and Laboratory Parameters and Side Effects

When the effect of Upadacitinib on laboratory parameters was evaluated, it was found that it did not cause clinically significant changes in glucose, HbA1c, AST, ALT, ALP, GGT, total bilirubin, creatinine, TSH, or FT4 levels (*p* > 0.05). A single instance of transient ALT elevation (179 U/L) was observed in one patient at month 3; however, this value returned to the normal range during follow-up. A statistically significant increase was observed in albumin and calcium levels, while decreases were observed in vitamin B12, folic acid, and ferritin levels (*p* < 0.05). While a decrease was observed in platelet, leukocyte, and neutrophil levels, lymphocyte and hemoglobin levels remained constant (Table 5). No hemogram change that fell to a critical level (e.g., neutrophils < 500/µL) was observed in any patient. A significant reduction was observed in both FCAL and CRP levels at the third and sixth months (*p* < 0.001).

**Figure 3:**
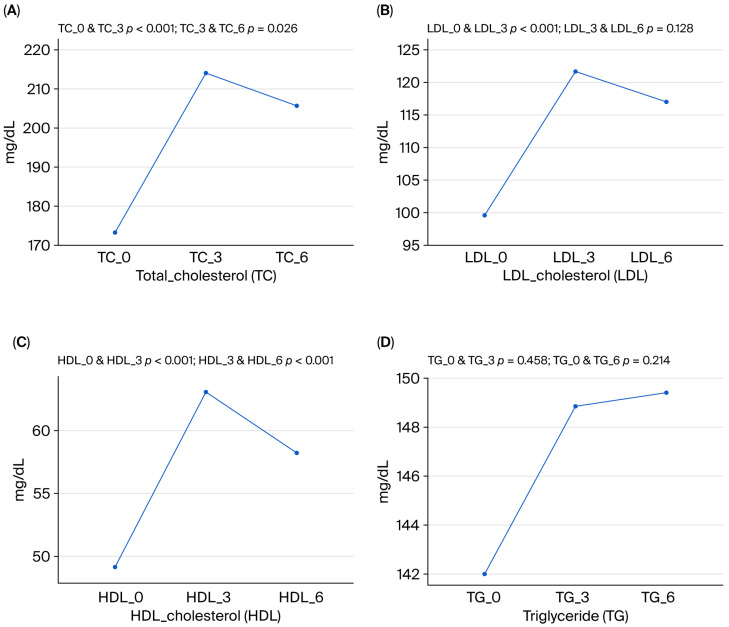
The effect of Upadacitinib treatment on lipid parameters. Levels are shown before Upadacitinib treatment (0) and at 3 and 6 months. Changes in (**A**) total cholesterol, (**B**) LDL cholesterol, (**C**) HDL cholesterol, and (**D**) triglyceride levels are presented.

Upadacitinib increased total cholesterol (TC), LDL-C, and HDL-C levels, but did not change triglyceride levels or the LDL-C/HDL-C ratio. Increases in TC, LDL-C, and HDL-C levels peaked in the third month of treatment. Compared to month 3, a partial reduction was observed at month 6 (the decline in LDL-C was not statistically significant, while the decreases in TC and HDL-C levels were statistically significant). Despite this, these values remained significantly higher than pre-Upadacitinib values (Table 5, Figure 3).

During Upadacitinib use, one patient developed herpes zoster, and three developed acne. One patient developed urticaria. A perianal abscess developed in one patient at the end of month 6, requiring inpatient treatment. This patient’s medication was changed due to a loss of efficacy. In addition to this case, three non-serious upper respiratory tract infections, one case of pneumonia, one case of cystitis, and one case of vaginitis were reported in outpatients. Two patients experienced severe side effects, such as nausea and vomiting, during induction therapy that prevented them from taking the medication, and these two patients were withdrawn from this study. Some patients also experienced non-serious symptoms such as nausea, vomiting, headache, and myalgia, which did not impair their quality of life (Table 6). No major adverse cardiovascular events (MACEs), such as embolism or acute coronary syndrome, were observed in any of our patients, and no deaths occurred.

## 4. Discussion

Upadacitinib is a more selective JAK inhibitor that has recently been used in both UC and CD patients. Both phase studies and limited real-world data have demonstrated its efficacy. Other JAK inhibitors have been used for many years in the treatment of various diseases, and some studies have highlighted their safety issues and side effect profiles. We aimed to investigate the effects of Upadacitinib on efficacy, side effects, and laboratory parameters in biologic-experienced IBD patients, 46.3% of whom had previously used more than one biologic.

Among our fully biologic-experienced UC patients, 40.8% had previously used more than one biologic. Of these patients, 31.8% had left-sided colitis, and 72.2% had extensive or pancolitis. For maintenance therapy, 90.9% of our patients received 30 mg of Upadacitinib. At the end of the sixth month, we found a persistence rate of 88%, a clinical response rate of 83.3%, and a clinical remission rate of 58.3% in UC patients. Pre-treatment FCAL levels were 521 ± 246 µg/g, and CRP was 74 ± 55 mg/L. At the end of the sixth month, 81.8% of patients had FCAL < 250 µg/g and CRP < 5 mg/L. Our clinical remission rate after 8 weeks of induction therapy was 45.5%. In phase 3 studies (U-ACHIEVE induction [UC1] and U-ACCOMPLISH [UC2]), the clinical remission rates were 26% and 34%, respectively, in induction therapy, while the clinical response rate according to the PMS was 79%. In the maintenance study (U-ACHIEVE maintenance [UC3]), the clinical remission rate at the end of week 52 was 51.7% in the Upadacitinib 30 mg arm (61.2% according to the PMS) [3]. The use of the PMS in our study may have contributed to these higher rates. In a study evaluating 8 weeks of induction therapy, high rates of clinical response (85.2%) and remission (81.2%) were observed [14], while in a study comparing JAK inhibitors, Upadacitinib persistence over 6 months was found to be 72.8% (95% CI [61.8–85.7]) [15].

Among our CD patients who had experienced at least one biologic, 52.7% had previously received more than one advanced therapy. The most common sites of involvement were the ileum (53.6%) and the ileocolon (31.6%). Regarding disease behavior, 31.6% of patients had perianal involvement (P), 63.2% had inflammatory disease (B1), and 21.1% had penetrating disease (B3). For maintenance therapy, 89.5% of our patients received 30 mg of Upadacitinib. At the end of the sixth month, we found a persistence rate of 78.3%, a clinical response rate of 72.7%, and a clinical remission rate of 50% in our CD patients. The pre-treatment fecal calprotectin (FCAL) level was 257 ± 198 µg/g, and that of CRP was 23 ± 16 mg/L. At the end of the sixth month, the proportion of patients with FCAL levels above 250 µg/g and CRP levels exceeding 5 mg/L was 10.2%. Our clinical remission rate during 12 weeks of induction therapy was 47.4%. In phase 3 studies (U-EXCEL and U-EXCEED), the clinical remission rates during induction therapy were found to be 49.5% and 38.9%, respectively [2]. In the maintenance study (U-ENDURE), at week 52, the clinical remission rate was 47.6% in the 30 mg Upadacitinib arm (2). In another CD study with 8 weeks of induction therapy, the clinical response and remission rates were found to be as high as 76.5% and 70.6%, respectively (14). Another multicenter retrospective study reported a 24-week persistence rate of 81.7%. The study also found that 64% of patients in the induction group achieved clinical remission, while the remission rates for CRP and FCAL were 55% and 50%, respectively [4].

In our study, Upadacitinib increased total cholesterol (TC), LDL-C, and HDL-C levels, but did not change triglyceride levels or the LDL-C/HDL-C ratio. The increases in TC, LDL-C, and HDL-C levels showed a partial decrease in the sixth month of treatment compared to the third month. Still, these values remained significantly higher than pre-Upadacitinib values. Studies in IBD patients have found that blood lipid levels may decrease during periods of inflammation [16]. In studies of UC patients using Tofacitinib, an increase in serum lipid levels (especially TC, HDL-C, and LDL-C) was reported within 4–8 weeks. These levels remained stable during follow-up and returned to baseline upon drug discontinuation [17,18]. Phase studies evaluating the use of Upadacitinib in both RA and UC patients have reported a moderate increase in lipid levels, while the LDL-C/HDL-C ratio remained unchanged [3,19]. A meta-analysis evaluating the effect of Upadacitinib on lipid profile and cardiovascular events from 15 placebo-controlled RCTs (*n* = 7695) reported that Upadacitinib at a dose of 15 mg increased LDL-C by 15.18 mg/dL (95% CI: 7.77–22.59) and HDL-C by 7.89 mg/dL (95% CI: 7.08–8.69), but Upadacitinib had no effect on MACEs (risk ratio (RR): 0.62; 95% CI: 0.24–1.60) [20]. While our study found that Upadacitinib increases cholesterol levels, our short follow-up period does not allow for a direct analysis of long-term cardiovascular risk.

In our study, Upadacitinib did not cause a significant change in glucose, HbA1c, AST, ALT, ALP, GGT, total bilirubin, creatinine, TSH, or FT4 levels (*p* > 0.05). Transient ALT elevation (179 U/L) was observed in one patient at 3 months, which spontaneously resolved during follow-up. A statistically significant increase was observed in albumin and calcium levels, while decreases were observed in vitamin B12, folic acid, and ferritin levels (*p* < 0.05). In a study conducted with other JAK inhibitors in rheumatoid arthritis patients, Tofacitinib did not affect HbA1c, but Baricitinib decreased HbA1c [21]. This study is among the rare investigations conducted on patients with IBD to evaluate the effect of Upadacitinib on glucose levels. We found that the drug had no effect on HbA1c and fasting blood sugar. A Chinese multicenter study involving 236 patients (156 with CD, 80 with UC) found that Upadacitinib treatment improved albumin levels and the proportion of patients with normal hemoglobin. In contrast, other laboratory parameters, including platelets, liver function tests, and renal function markers, remained within normal ranges throughout treatment [22]. The improvement in albumin levels was not attributed to Upadacitinib but rather to decreased inflammation and optimal nutritional status. In phase studies such as U-ENDURE and U-ACCOMPLISH, increases in liver function tests were found to be greater in those receiving long-term treatment (maintenance therapy rather than induction therapy), with rates ranging from 2.5% to 10%. The incidence of renal impairment in these phase studies was less than 1% [2,3]. The absence or rarity of increases in liver function tests in our study and real-world studies may be due to the lack of studies with a comparable duration to phase studies. We were unable to find any previous studies examining the effects of Upadacitinib use on thyroid function tests (TFTs) such as FT4 and TSH in patients with IBD. Our study observed that Upadacitinib did not affect TFTs over 6 months.

In our study, a decrease in platelet, leukocyte, and neutrophil levels was observed, while lymphocyte and hemoglobin levels remained stable. No patient had a hemogram change that fell to critical levels (e.g., lymphocytes < 500/µL). In phase studies that included both induction and maintenance therapy, neutropenia was observed in 1.2% to 6% of patients receiving Upadacitinib, and lymphopenia in 1.4% to 6%; this rate was significantly higher compared to the placebo arm. In these studies, the rate of anemia was found to be lower in UC patients compared to the placebo arm. However, in CD patients, while no definitive conclusion could be drawn, the rate was generally slightly higher in the Upadacitinib arm [2,3]. A study evaluating the efficacy and safety of Tofacitinib in 4481 patients with RA over a 9.5-year period found the incidence rates (IRs) for neutropenia and lymphopenia to be 0.52 and 1.11, respectively. In this study, no patients experienced serious infection during the month of the lowest neutrophil count. Of the 58 cases with severe lymphopenia (<500 cells/mm^3^), 5 developed a serious infectious event [23]. They identified JAK2 signaling blockade, which interferes with hematopoiesis, as the culprit. In our study, the decrease in platelet and neutrophil counts appeared to be due to the suppression of inflammation rather than a direct drug effect.

The overall side effect rate in our patient cohort was 48.8%, with infections being the most common at 17.1%. The most frequent symptom was nausea and vomiting (9.8%), while one patient experienced herpes zoster, and three experienced acne (7.3%). Except for one perianal abscess that required inpatient treatment, all other patients were treated as outpatients and did not require drug discontinuation. No embolism, acute coronary syndrome, or death occurred in any of our patients. The high side effect rate in our study is likely attributed to our meticulous recording of even mild symptoms. We observed no serious side effects, unlike those reported in phase studies [2,3]. A 16-week study comparing Tofacitinib with Upadacitinib observed no venous thromboembolic events or serious infections, and our study showed similar results [24]. Most studies with real-world data have reported rates of serious side effects that are either similar to ours or less frequent than those in phase studies [4,14,15,22]. The low incidence of serious adverse events observed in this study and other real-world investigations is likely a result of the short follow-up, as most available data pertain to induction therapy.

### Study Limitations and Strengths

The small number of patients and the lack of long-term follow-up are the main weaknesses of our study. A significant limitation of our study is the absence of systematically collected data on endoscopic remission, despite its growing importance in IBD treatment. This is one of the few real-world studies outside of phase trials that includes both induction and short-term maintenance therapy. Furthermore, this study is the first to evaluate the effects of Upadacitinib on laboratory parameters in IBD, including glucose, HbA1c, and thyroid function tests (FT4 and TSH).

## 5. Conclusions

This real-world study confirms that Upadacitinib is an effective and well-tolerated treatment for biologic-experienced patients with moderate-to-severe IBD. We observed high rates of clinical response and remission within six months, supported by a significant reduction in key disease markers, including fecal calprotectin and C-reactive protein. The drug’s safety profile was favorable, with no serious adverse events, venous thromboembolisms, or major cardiovascular events reported. While Upadacitinib increased cholesterol levels, it did not demonstrate a negative effect on the LDL/HDL ratio or glucose metabolism. Our findings support the use of Upadacitinib in biologic-unresponsive IBD patients. However, further multicenter, longer-term studies are required to evaluate its long-term safety and efficacy.

## Figures and Tables

**Table 1:** Demographic data of patients before Upadacitinib treatment.

		Total Group (*n* = 41)	UC (*n* = 22)	CD (*n* = 19)
Gender (female)		36.6% (*n* = 15)	40.1% (*n* = 9)	31.6% (*n* = 6)
Age (years)		43.6 ± 14.3	48 ± 15.4	38.1 ± 10.7
Disease duration (years)		8.1 ± 5.1	7.8 ± 4.4	8.5 ± 5.9
Smoking		41.5% (*n* = 17)	36.4% (*n* = 8)	47.4% (*n* = 9)
Prior surgery		12.2% (*n* = 5)	0% (*n* = 0)	26.3% (*n* = 5)
1 Prior biologics		53.7% (*n* = 22)	59.1% (*n* = 13)	47.4% (*n* = 9)
>1 Prior biologics		46.3% (*n* = 19)	40.9% (*n* = 9)	53.7% (*n* = 10)
Location (CD)	L1(ileal)	-	-	53.7% (*n* = 10)
(Montreal classification)	L2 (colonic)	-	-	15.8% (*n* = 3)
	L3 (ileocolonic)	-	-	26.3% (*n* = 5)
	L4 (upper GIS)	-	-	5.3% (*n* = 1)
Behavior (CD)	B1	-	-	63.2% (*n* = 12)
(Montreal classification)	B2	-	-	26.3% (*n* = 5)
	B3	-	-	21.1% (*n* = 4)
Perianal disease		14.6% (*n* = 4)	0% (*n* = 0)	31.6% (*n* = 6)
Location (UC)	Left-sided	-	31.8% (*n* = 7)	-
	Extensive	-	40.9% (*n* = 9)	-
	Pancolitis	-	27.3% (*n* = 6)	-
EIM	Enteropathic arthritis	36.6% (*n* = 15)	22.7% (*n* = 5)	53.7% (*n* = 10)
	Dermatological	9.8% (*n* = 4)	13.6% (*n* = 3)	5.3% (*n* = 1)
	Ocular	4.9% (*n* = 2)	4.5% (*n* = 1)	5.3% (*n* = 1)
	PSC	2.4% (*n* = 1)	4.5% (*n* = 1)	0% (*n* = 0)
Ankylosing spondylitis		19.5% (*n* = 8)	4.5% (*n* = 1)	36.9% (*n* = 7)
Comorbidities	Diabetes	2.4% (*n* = 1)	4.5% (*n* = 1)	0% (*n* = 0)
	Hypertension	14.6% (*n* = 6)	13.6% (*n* = 3)	15.8% (*n* = 3)
	Respiratory	7.3% (*n* = 3)	9.1% (*n* = 2)	5.3% (*n* = 1)
	Renal disease	2.4% (*n* = 1)	4.5% (*n* = 1)	0% (*n* = 0)
	Other	12.2% (*n* = 5)	59.1% (*n* = 2)	15.8 (*n* = 3)
	Total	39% (*n* = 16)	40.9% (*n* = 9)	36.9% (*n* = 7)
Fecal calprotectin (μg/g)		405 ± 260	521 ± 246	257 ± 198
CRP (mg/L)		52 ± 49	74 ± 55	23 ± 16
Albumin (g/dL)		4 ± 0.6	3.9 ± 0.52	4.1 ± 0.36

Abbreviations: B1, non-stricturing, non-penetrating; B2, stricturing; B3, penetrating; CD, Crohn’s disease; EIM, extraintestinal manifestations; PSC, primary sclerosing cholangitis; UC, ulcerative colitis.

**Table 2:** Prior biologic and immunomodulator use in the study cohort.

	Total Group (*n* = 41) *n* (%)	UC (*n* = 22) *n* (%)	CD (*n* = 19) *n* (%)
Infliximab	26 (63.4%)	13 (59.1%)	13 (68.4%)
Adalimumab	22 (53.7%)	11 (50%)	11 (57.9%)
Vedolizumab	10 (24.4%)	5 (22.7%)	5 (26.3%)
Ustekinumab	9 (22%)	5 (22.7%)	4 (21.1%)
Two biological molecules	14 (34.1%)	7 (31.8%)	7 (36.9%)
Three biological molecules	3 (7.3%)	1 (4.5%)	2 (10.5%)
Four biological molecules	2 (4.9%)	1 (4.5%)	1 (5.3%)
Azathioprine	36 (87.8%)	19 (86.4%)	17 (89.5%)
Methotrexate	2 (4.9%)	0 (0%)	2 (10.5%)
Steroid + UPA	11 (26.8%)	5 (22.7%)	6 (31.6%)

Abbreviations: CD, Crohn’s disease; Steroid + UPA, corticosteroids were started concurrently with Upadacitinib; UC, ulcerative colitis.

**Table 3:** Partial Mayo score, FCAL, and CRP course in UC patients receiving Upadacitinib treatment.

Ulcerative Colitis (*n* = 22)				
		Baseline *n* (%)	3rd Month of UPA *n* (%)	6th Month of UPA *n* (%)
PMS	<2 (remission)	0 (0%)	10 (45.5%)	14 (63.6%)
	2–4 (mild)	2 (9.1%)	9 (40.1%)	6 (27.3%)
	5–7 (moderate)	8 (36.4%)	3 (13.6%)	1 (4.5%)
	>7 (severe)	12 (54.5%)	0 (0%)	1 (4.5%)
FCAL (µg/g)	>250	18 (81.8%)	5 (22.7%)	4 (18.2%)
	100–250	4 (18.2%)	8 (36.4%)	9 (40.1%)
	<100	0 (0%)	9 (40.1%)	9 (40.1%)
CRP (mg/L)	>5	22 (100%)	7 (31.8%)	4 (18.2%)
	≤5	0 (0%)	15 (68.2%)	18 (81.8%)

Abbreviations: CRP, C-reactive protein; FCAL, fecal calprotectin; PMS, partial Mayo score; UPA, Upadacitinib.

**Table 4:** Harvey–Bradshaw Index, FCAL, and CRP course in CD patients receiving Upadacitinib treatment.

Crohn Disease (*n* = 19)				
		Baseline *n* (%)	3rd Month of UPA *n* (%)	6th Month of UPA *n* (%)
HBI	<5 (remission)	0 (0%)	9 (47.4%)	11 (57.9%)
	5–7 (mild)	5 (26.3%)	4 (21.1%)	3 (15.8%)
	8–16 (moderate)	11 (57.9%)	6 (31.6%)	5 (26.3%)
	>16 (severe)	3 (15.8%)	0 (0%)	0 (0%)
FCAL (µg/g)	>250	8 (42.1%)	4 (21.1%)	2 (10.5%)
	100–250	8 (42.1%)	4 (21.1%)	6 (31.6%)
	<100	3 (15.8%)	11 (57.9%)	11 (57.9%)
CRP (mg/L)	>5	18 (94.7%)	11 (57.9%)	10 (52.6%)
	≤5	1 (5.3%)	8 (42.1%)	9 (47.4%)

Abbreviations: CRP, C-reactive protein; FCAL, fecal calprotectin; HBI, Harvey–Bradshaw Index; UPA, Upadacitinib.

**Table 5:** Laboratory values at baseline, 3, and 6 months of Upadacitinib treatment.

All Patients (*n* = 41)	Baseline (Mean ± Std)	Month 3 of UPA (Mean ± Std)	Month 6 of UPA (Mean ± Std)	*p*-Value
Glucose (mg/dL)	93.3 ± 18.9	92.4 ± 19.2	90.8 ± 11.7	0.289
HbA1c (%)	5.27 ± 0.75	5.28 ± 0.86	5.26 ± 0.79	0.914
Total-C (mg/dL)	173.3 ± 41.8	214.1 ± 50.1	205.6 ± 52.7	0.001
LDL-C (mg/dL)	99.6 ± 35.0	121.7 ± 40.8	117.0 ± 42.5	0.000
HDL-C (mg/dL)	49.1 ± 13.9	63.1 ± 17.1	58.2 ± 15.0	0.000
LDL-C/HDL-C (ratio)	2.09 ± 0.68	2.00 ± 0.69	2.08 ± 0.74	0.233
Triglyceride(mg/dL)	142.0 ± 75.0	148.9 ± 86.6	149.4 ± 79.4	0.542
AST (U/L)	20.5 ± 7.9	23.4 ± 11.0	21.3 ± 7.1	0.429
ALT (U/L)	21.1 ± 14.6	29 ± 29.6	22.5 ± 14.3	0.076
ALP (U/L)	76.5 ± 19.9	76.3 ± 20.9	74.2 ± 21.1	0.433
GGT (U/L)	22.4 ± 10.9	21.9 ± 13.2	19.3 ± 9.3	0.076
Total bilirubin (mg/dL)	0.55 ± 0.29	0.58 ± 0.30	0.56 ± 0.30	0.614
Albumin (g/dl)	4.01 ± 0.46	4.34 ± 0.38	4.37 ± 0.36	0.000
Calcium (mg/dL)	9.21 ± 0.43	9.46 ± 0.55	9.43 ± 0.52	0.002
Creatinine (mg/dL)	0.78 ± 0.22	0.78 ± 0.22	0.81 ± 0.26	0.502
TSH (µIU/mL)	1.74 ± 0.85	1.70 ± 0.77	1.55 ± 0.73	0.059
FT4 (pmol/L)	11.2 ± 2.1	10.8 ± 1.43	10.8 ± 1.24	0.072
Vit_B12 (pg/mL)	267 ± 112	230 ± 81	218 ± 72	0.000
Folic acid (ng/mL)	8.18 ± 3.08	7.57 ± 3.29	7.28 ± 3.27	0.016
Ferritin (ng/mL)	80.8 ± 118	53.4 ± 77.1	50.7 ± 73.6	0.001
Hgb (g/dL)	13.0 ± 1.40	13.2 ± 1.24	13.1 ± 2.51	0.689
Plt (×10^3^/µL)	348 ± 93.2	320 ± 88.4	302 ± 86.6	0.000
WBC (×/µL)	9747 ± 2820	8161 ± 2699	7818 ± 2451	0.000
Neutrophil (×/µL)	7199 ± 5577	4934 ± 2260	4975 ± 2166	0.012
Lymphocyte (×/µL)	2436 ± 1358	2314 ± 944	2122 ± 719	0.118
CRP (mg/L)	29.8 ± 31.5	8.56 ± 13.3	7.49 ± 11.23	0.000
FCAL (µg/g)	405 ± 259	158 ± 158	120 ± 112	0.000

Abbreviations: ALP, Alkaline Phosphatase; ALT, Alanine Aminotransferase; AST, Aspartate Aminotransferase; CRP, C-reactive protein; FCAL, fecal calprotectin; FT4, Free Thyroxine; GGT, Gamma-Glutamyl Transferase; HbA1c, Hemoglobin A1c; HDL-C, High-Density Lipoprotein Cholesterol; Hgb, Hemoglobin; LDL-C, Low-Density Lipoprotein Cholesterol; Plt, Platelet; TSH, Thyroid-Stimulating Hormone; Total-C, Total Cholesterol; UPA, Upadacitinib; Vit_B12, Vitamin B12; WBC, White Blood Cell.

**Table 6:** Side effects that developed during Upadacitinib treatment.

Adverse Events		All Patients (*n* = 41) *n* (%)
Infection	Upper respiratory tract infection	3 (7.3%)
	Pneumonia	1 (2.4%)
	Perianal abscess	1 (2.4%)
	Other	2 (4.9%)
Dermatological	Herpes zoster reactivation	1 (2.4%)
	Acne	3 (7.3%)
	Urticaria	1 (2.4%)
Symptoms	Nausea and vomiting	4 (9.8%)
	Headache	2 (4.9%)
	Myalgia	2 (4.9%)
Total		20 (48.8%)

## Data Availability

The datasets generated and/or analyzed during the current study are presented in the manuscript.

## Abbreviations

CD	Crohn’s disease
CRP	C-reactive protein
FCAL	Fecal calprotectin
GIS	Gastrointestinal system
HBI	Harvey–Bradshaw Index
IBD	Inflammatory bowel disease
JAK	Janus kinase
MACE	Major adverse cardiac events
PMS	Partial Mayo score
PSC	Primary sclerosing cholangitis
TC	Total cholesterol
UC	Ulcerative colitis
UPA	Upadacitinib
